# Role of *CD152* genetic polymorphisms in the susceptibility to breast cancer

**DOI:** 10.18632/oncotarget.15794

**Published:** 2017-03-01

**Authors:** Hai Chen, Xiaodong Qi, Xue Bai, Ping Qiu, Bin Chen

**Affiliations:** ^1^ Department of Galactophore, The General Hospital of Beijing Military Command, Beijing 100000, China

**Keywords:** CD152, CTLA-4, polymorphisms, breast cancer, immune response

## Abstract

**Background:**

The polymorphisms in cluster of differentiation 152 (*CD152*) gene have been reported to be associated with breast cancer (BC), but relevant findings were far from conclusive. Therefore, we carried out this meta-analysis to combine those results for a clearer perspective on this issue.

**Results:**

In our meta-analysis, a total of 8 eligible publications of 19 case-control studies were selected, which totally contained 7,442 BC cases and 7,376 normal controls. Among the five polymorphisms of *CD152* gene, +49 G/A, −1661 A/G and −318 C/T significantly increased the risk of BC under corresponding genetic comparisons; while CT60 G/A polymorphism was negatively related to the cancer susceptibility. In addition, −1772 T/C polymorphism of *CD152* gene was not associated with the development of BC.

**Materials and methods:**

Online databases and other sources were searched for published studies on the relationship between BC susceptibility and *CD152* polymorphisms (+49 G/A, −1661 A/G, −1722 T/C, −318 C/T and CT60 G/A). The strength of association was evaluated with pooled odds ratios (ORs) and their corresponding 95% confidence intervals (95% CIs). Heterogeneity evaluation was conducted via *Q* test. Sensitivity analysis was used to detect the stability of our results. Begg's funnel plot and Egger's test were applied to investigate publication bias among selected studies.

**Conclusions:**

The polymorphisms +49 G/A, −1661 A/G and −318 C/T may elevate the susceptibility to BC, but the polymorphism CT60 G/A may offer protection against the cancer.

## INTRODUCTION

Breast cancer (BC) is the most commonly diagnosed malignancy and a leading cause of cancer-related deaths in women around the world [1, 2]. The global incidence rate of BC is about 13%, and shows an upward tendency in both developing and developed countries and regions [3, 4]. The exact mechanism of breast cancer initiation is still beyond totally understood, though many factors have been identified to affect the pathogenesis of this malignancy. Studies have pointed out that the development of BC is affected by multiple epidemiological factors, such as age, female reproductive status, short period of or no breast feeding, use of oral contraceptive, and previous benign breast disease [5, 6]. In addition, some environmental factors, such as chemical carcinogens and ionizing radiation, have also been proposed to contribute to increased risk of BC [7]. However, only a few of people exposed to these factors develop BC, indicating the important role of genetic factors [8, 9].

Cluster of differentiation 152 (CD152), also know as cytotoxic T-lymphocyte-associated protein 4 (CTLA-4), is a homologue of CD28, and functions as an inhibitor receptor for B7 which is a co-stimulatory molecule on mature antigen-presenting cells [10, 11]. CD152 acts as a negative regulator of T cells involved in antitumor immune responses [12], and its blockade can promote immune responses [13] and reject tumors [14]. The hypothesis has been put forward that CD152 may attenuate the antitumor responses and increase cancer susceptibility via elevating the activation threshold of T cells in early stage of tumorigenesis [15]. Human *CD152* gene is located on chromosome 2q33, and contains 4 exons coding for a leader sequence, an extracellular domain, a transmembrane domain and a cytoplasmic tail [16]. As a highly polymorphic gene, *CD152* has been identified to possess numerous polymorphisms, including −1661A/G, −1722T/C, −318C/T, +49A > G and CT60G/A [17]. These polymorphisms are functionally important, because they may affect immune responses via changing CD152 expression levels and functions on T cells. Specifically, they may alter the transcription capacity of the *CD152* gene [18], the processing and transport of CD152 protein [19], and the interactions between CD152 and CD80 ligand [20]. Therefore, they have been widely explored their influences on autoimmune disorders [21] and cancer [22].

The polymorphisms +49 G/A (rs231775), −1661 A/G (rs4553808), −1722 T/C (rs733618), −318 C/T (rs5742909) and CT60 G/A (rs3087243) in the *CD152* gene have been discussed their relationship with the susceptibility to BC in previous studies, but relevant findings were conflicting. So we performed this meta-analysis to pool these results for a more comprehensive conclusion.

## RESULTS

### Study characteristics

**Figure 1:**
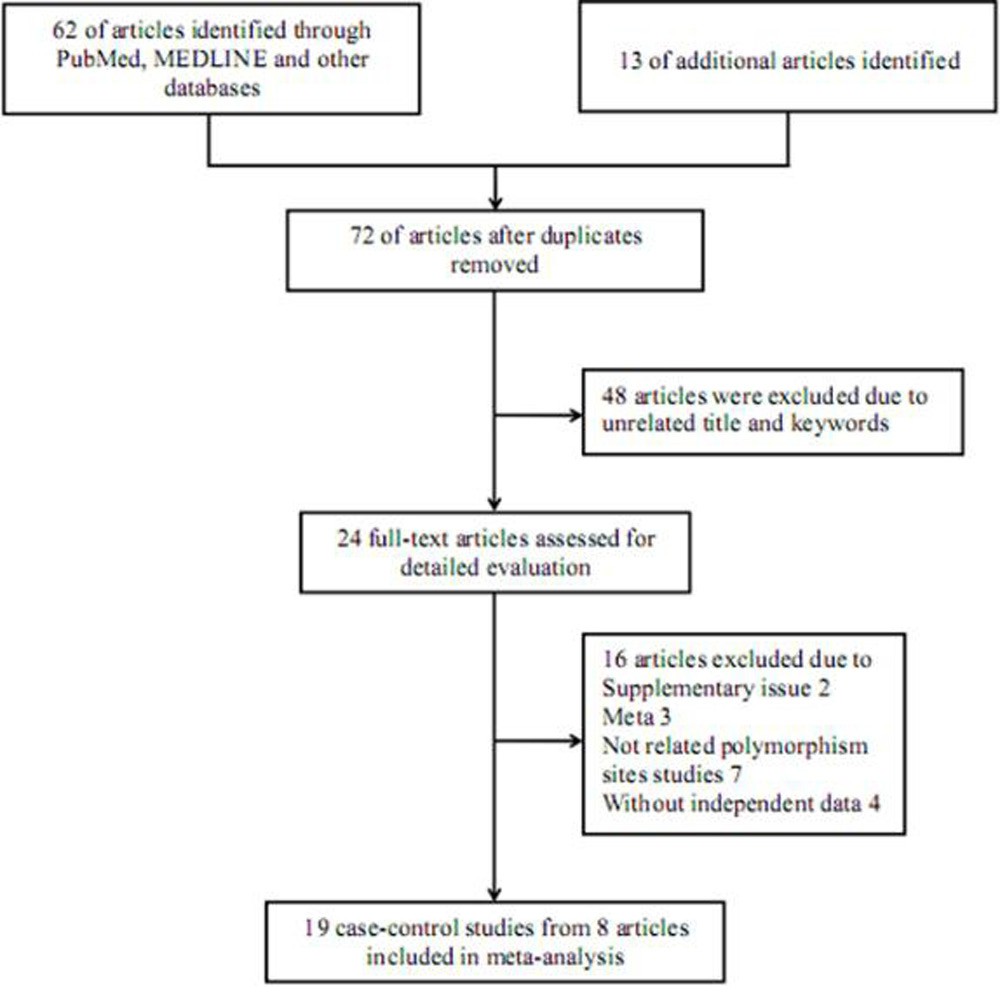
Flowchart of literature selection with detailed reasons for exclusion

Altogether, 75 potentially relevant publications were initially retrieved using the searching strategy. After strict screening, 67 of them were excluded for not conforming to the criteria above mentioned (Figure 1), and finally, 8 eligible articles containing 19 case-control studies were incorporated into the present meta-analysis [23–30]. All of these studies were carried out in Asian populations, with population-based controls. Table 1 displays main information of each selected study.

**Table 1:** Main information of studies included in the meta-analysis

SNP	First author	Year	Ethnicity (Country)	Genotyping method	Case			Control			Control source	HWE
+49G/A					GG	GA	AA	GG	GA	AA		
	Ghaderi	2004	Asian (Iran)	PCR-RFLP	9	104	84	19	72	60	PB	0.717
	Wang	2007	Asian (China)	PCR-RFLP	10	59	48	23	70	55	PB	0.926
	Minhas	2014	Asian (India)	PCR-RFLP	26	113	111	24	121	105	PB	0.197
	Li	2012	Asian (China)	PCR-RFLP	246	281	49	256	243	54	PB	0.739
	Sun	2008	Asian (China)	PCR-RFLP	474	485	101	559	446	65	PB	0.052
	Sun	2008	Asian (China)	PCR-RFLP	482	455	100	546	451	73	PB	0.118
−1661A/G					AA	AG	GG	AA	AG	GG		
	Wang	2007	Asian (China)	PCR-RFLP	62	45	2	111	35	2	PB	0.683
	Kong	2010	Asian (China)	PCR-RFLP	204	105	6	241	76	5	PB	0.721
	Erfani	2006	Asian (Iran)	PCR-RFLP	211	65	6	184	43	11	PB	0.0003
	Li	2012	Asian (China)	PCR-RFLP	405	153	16	425	115	11	PB	0.331
−1722T/C					TT	TC	CC	TT	TC	CC		
	Li	2008	Asian (China)	PCR-RFLP	125	163	40	111	168	48	PB	0.225
	Li	2012	Asian (China)	PCR-RFLP	184	276	114	207	256	88	PB	0.552
	Erfani	2006	Asian (Iran)	PCR-CTPP	225	54	3	204	41	0	PB	0.153
−318C/T					CC	CT	TT	CC	CT	TT		
	Wang	2007	Asian (China)	PCR-RFLP	84	33	0	129	19	0	PB	0.404
	Kong	2010	Asian (China)	PCR-RFLP	225	83	7	263	54	5	PB	0.257
	Erfani	2006	Asian (Iran)	PCR-ARMS	244	38	1	206	31	4	PB	0.036
CT60 G/A					GG	GA	AA	GG	GA	AA		
	Wang	2007	Asian (China)	PCR-RFLP	24	47	46	18	56	74	PB	0.155
	Li	2008	Asian (China)	PCR-RFLP	32	124	172	20	114	193	PB	0.566
	Li	2012	Asian (China)	PCR-RFLP	361	197	23	361	182	23	PB	0.992

Notes: PCR-RFLP, polymerase chain reaction-restriction fragment length polymorphism; PCR-CTPP, polymerase chain reaction with confronting two pairs primers; PCR-ARMS, PCR-amplification refractory mutation system; HWE, Hardy-Weinberg equilibrium.

### Meta-analysis results

**Figure 2:**
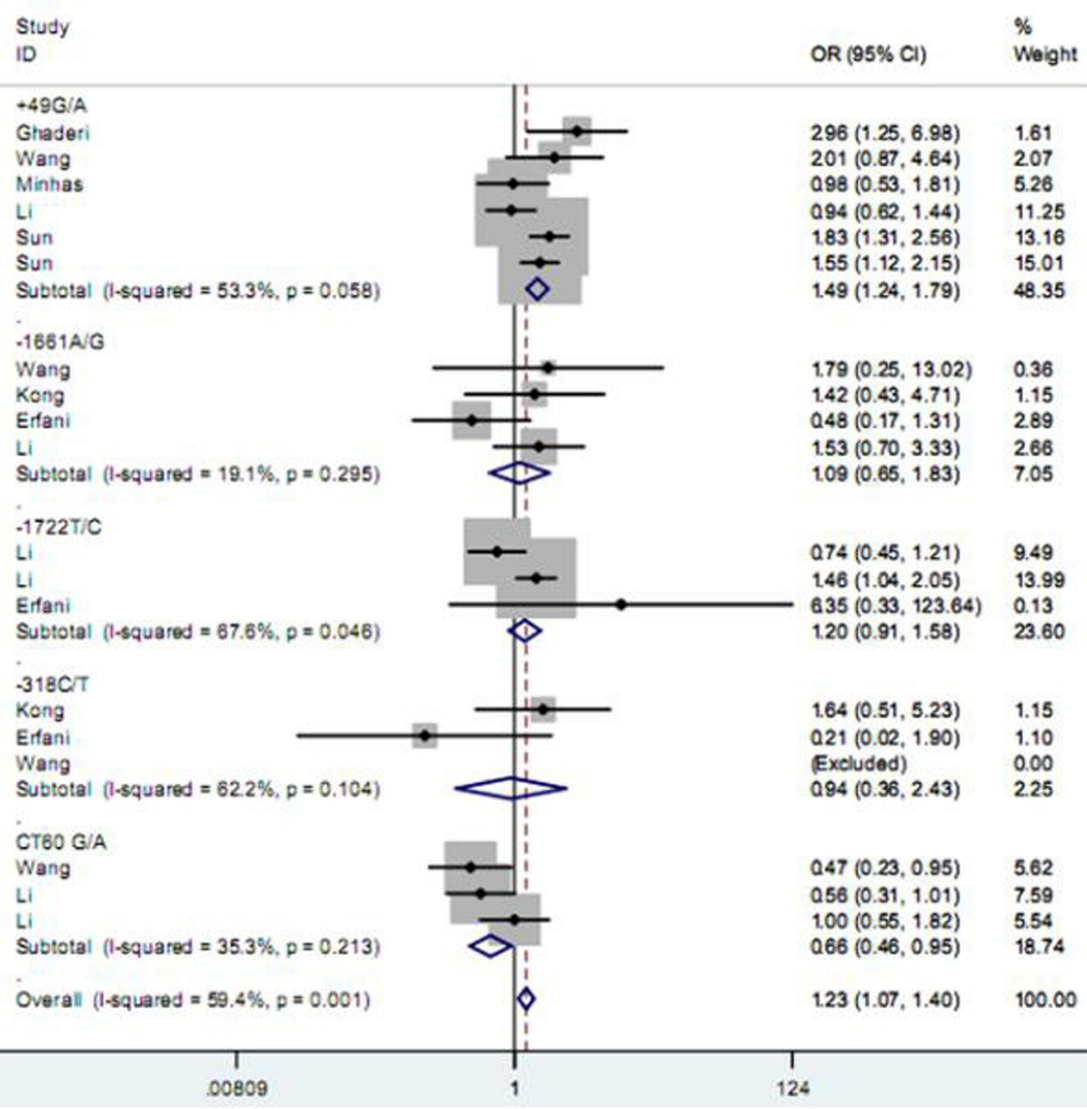
Forest plot for the association between *CD152* polymorphisms and breast cancer susceptibility under homozygote model

**Figure 3:**
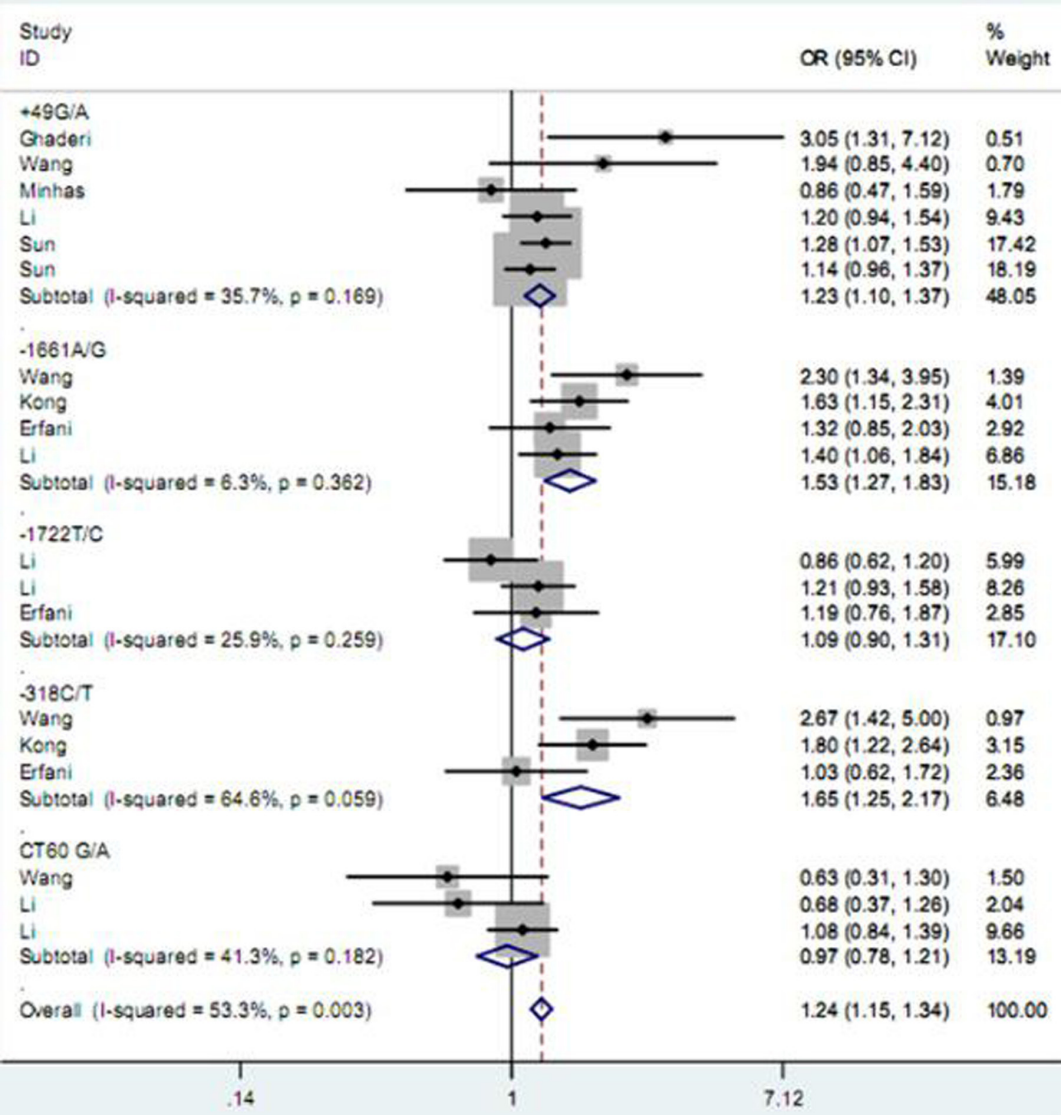
Forest plot for the association between *CD152* polymorphisms and breast cancer susceptibility under heterozygote model

As described in Table 2, among five studied polymorphisms in *CD152* gene, three of them were related to increased risk of BC and one was negatively associated with the cancer susceptibility, while the other one showed no significant relationship under any one of genetic contrasts. Specifically, the polymorphism +49 G/A elevated the risk of BC under all five contrasts of AA vs. GG (Figure 2), AA+GA vs. GG, AA vs. GG + GA, A vs. G and GA vs. GG (Figure 3) (OR = 1.49, 95% CI = 1.24–1.79; OR = 1.27, 95% CI = 1.14–1.40; OR = 1.25, 95% CI = 1.08–1.46; OR = 1.19, 95% CI = 1.11–1.29; OR = 1.23, 95% CI = 1.10–1.37); the −1661 A/G polymorphism enhanced the BC susceptibility under GG+AG vs. AA, G vs. A and AG vs. AA (Figure 3) genetic models; and the -318 C/T polymorphism increased the BC risk under CT vs. CC comparison (Figure 3); while the polymorphism CT60 G/A expressed a reducing effect on BC susceptibility under AA vs. GG (Figure 2) and AA vs. GG+GA contrasts. As for the polymorphism −1772 T/C, it showed no significant correlation with the susceptibility to BC.

**Table 2:** *CD152* polymorphisms and breast cancer susceptibility

Comparison	Odds ratio (95% confidence interval)/*P* value for heterogeneity											
	+49G/A		−1661A/G		−1722T/C		−318C/T		CT60 G/A		Total	
22 versus 11	1.49 (1.24, 1.79)	0.058	1.09 (0.65, 1.83)	0.295	1.15 (0.60, 2.22)	0.046	0.94 (0.36, 2.43)	0.104	0.66 (0.46, 0.95)	0.213	1.14 (0.89, 1.45)	0.001
22 + 12 versus 11	1.27 (1.14, 1.40)	0.148	1.48 (1.24, 1.77)	0.218	1.12 (0.93, 1.33)	0.100	1.61 (0.94, 2.78)	0.026	0.76 (0.46, 1.23)	0.047	1.26 (1.10, 1.43)	0.000
22 versus 11+12	1.25 (1.08, 1.46)	0.185	0.99 (0.59, 1.66)	0.337	1.14 (0.89, 1.47)	0.121	0.86 (0.33, 2.23)	0.126	0.77 (0.60, 0.97)	0.583	1.06 (0.90, 1.25)	0.025
2 versus 1	1.19 (1.11, 1.29)	0.424	1.36 (1.16, 1.59)	0.154	1.09 (0.85, 1.41)	0.044	1.48 (0.87, 2.51)	0.017	0.83 (0.63, 1.09)	0.038	1.15 (1.04, 1.28)	0.000
12 versus 11	1.23 (1.10, 1.37)	0.169	1.53 (1.27, 1.83)	0.362	1.09 (0.90, 1.31)	0.259	1.65 (1.25, 2.17)	0.059	0.97 (0.78, 1.21)	0.182	1.27 (1.12, 1.43)	0.003

Notes: 11, wild homozygote; 12, heterozygote; 22, rare homozygote; 1, wild allele; 2, rare allele.

### Heterogeneity test

*Q* test revealed no significant heterogeneity for the two polymorphisms of +49 G/A and −1661 A/G under any genetic comparisons, so the fixed-effects model was chosen for ORs calculation. As for the other three polymorphisms, the choice of which model being utilized was determined according to the standard above described.

### Sensitivity analysis

Sensitivity analysis was performed for the polymorphisms +49 G/A and −1661 A/G, and no substantial alteration occurred during this process, indicating the final results were statistically robust. When it came to the other three polymorphisms, such analysis was not carried out for them due to limited number of included studies for each polymorphism.

### Publication bias investigation

**Figure 4:**
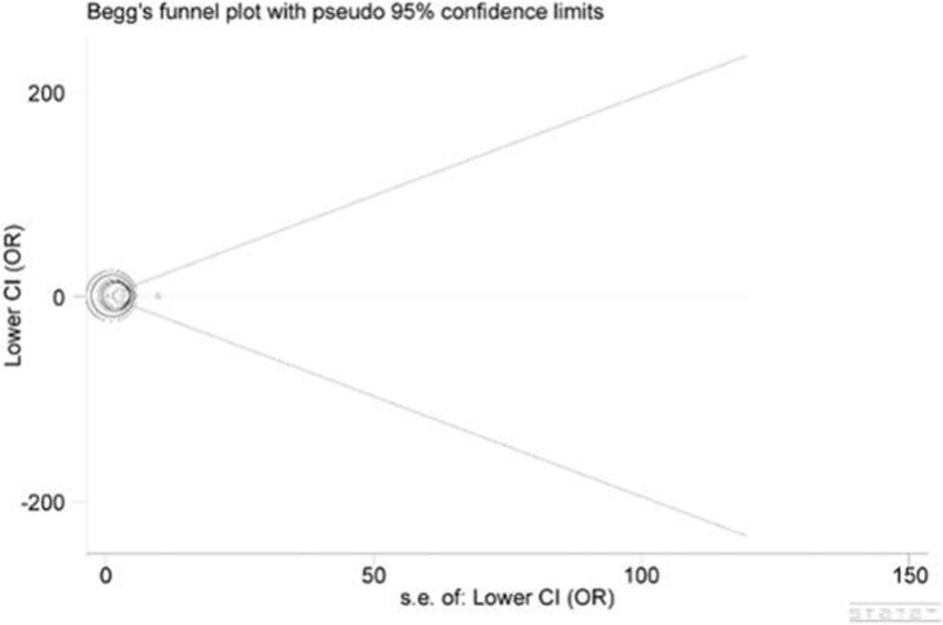
Begg's funnel plot for publication bias

Begg's funnel plot and Egger's test were employed to inspect publication bias across included studies. As a result, the shape of funnel plots seemed symmetrical (Figure 4), implying publication bias was negligible. Furthermore, these implications were all confirmed by statistical evidence from Egger's test (*P* = 0.447).

## DISCUSSION

BC is the most frequent malignancy in women all over the world. The incidence rate of BC in China is lower than that in western countries, but it still shows a rising trend in the past few years, especially in those big coastal cities of this country. With complex pathogenesis, multiple factors may contribute to the occurrence and development of BC. Many elements have been identified as potential risk factors for BC onset, such as positive familial history of BC, history of benign breast diseases, excessive exposure to ionizing radiation, early menarche, alcohol consumption, and excessive intake of saturated fatty acid and meat. However, among people exposing to the same environment and having similar lifestyle, only a part of them develop to BC. Although the initiation and progression of the majority of human cancers have been shown to be the results of combined actions between environmental and genetic factors, some studies demonstrate that heredity contributes to about one fourth of all BC cases. All these phenomenons imply that genetic factors play a critical role in the initiation of BC.

As one of the most fundamental immunosuppressive cytokines, CD152 is mainly expressed on activated T cells. It functions as a restraining regulator for the proliferation and activation of T cells, further inhibiting the immune functions of T cells through inducing Fas-independent apoptosis of activated T cells. Additionally, CD152 is also expressed on different cellular types, both normal and neoplastic. CD152 can suppress cell-cycle progression through inhibiting interleukin-2 production, thus resulting in the induction and maintenance of T cell tolerance. The CD152 molecule can reduce the responses of T cells to foreign antigens and autoantigens under physiological conditions. It is up-regulated on the surface of the latter in tumor microenvironment. Besides, CD152 deficiency can lead to lethal diseases, including cancer. *CD152* gene consists of 4 exons and 3 introns, with more than 100 polymorphisms. These polymorphisms may alter the expression and/or the activity of the protein, thus being involved in the etiology of multiple diseases.

In the previous tumor investigations, a number of studies were carried out to estimate the association between the polymorphisms (+49 G/A, −1661 A/G, −1722 T/C, −318 C/T and CT60 G/A) of *CD152* gene and the susceptibility to BC. Wang et al. found that the −1661 A/G polymorphism G allele, the −318 C/T polymorphism T allele and the CT60 G/A polymorphism G allele were more frequent in BC patients than that in controls. They concluded that these alleles increased the risk of the cancer, while the +49G/A polymorphism was only associated with tumor size in the patients [25]. For −1661 A/G polymorphism, Li et al. got a similar result as Wang et al., but they insisted there was no significant correlation of BC with either the +49 G/A or CT60 G/A polymorphism. Nevertheless, they put forward that the CC genotype and C allele of the −1722 T/C polymorphism increased the risk of the cancer [26]. However, Erfani et al. revealed no significant difference of genotype or allele frequencies of −1722 T/C, −1661 A/G and −318C/T polymorphisms between BC patients and healthy controls in Indians [27].

Based on Asian populations, these studies got no consistent opinion on the relationship between *CD152* polymorphisms and BC susceptibility, which is the reason for us to perform this meta-analysis. Pooled analysis demonstrated that the +49 G/A, −1661 A/G and −318 C/T polymorphisms could increase the risk of BC, while CT60 G/A polymorphism exerted an opposite function on the susceptibility to the malignancy. Meanwhile, −1722 T/C polymorphism did not show significant link with the cancer developing. The results were obtained based on strict analyses, but they still need to be identified by studies with larger sample sizes, due to some limitations in this meta-analysis. Firstly, the number of included studies was small, which might affect the comprehensiveness of the final outcomes. Secondly, subgroup analysis was not performed in the present study due to limited data. Thirdly, as we all know, BC was a complicated diseases caused by multiple factors and interactions among them, but this respect was not explored in our meta-analysis.

In summary, the +49 G/A, −1661 A/G and −318 C/T polymorphisms of the *CD152* gene have a positive relationship with BC susceptibility while the CT60 G/A polymorphism is negatively related to the cancer.

## MATERIALS AND METHODS

### Literature searching strategy

The electronic databases of PubMed, EMBASE, Google Scholar Web, CNKI and Wanfang were searched for studies on the association between *CD152* polymorphisms and BC risk, using the combination of key terms as followed: “Cluster of differentiation 152′′ or “CD152” or “cytotoxic T-lymphocyte-associated protein 4” or “CTLA-4′′ or “ALPS5” or “GSE”, “breast cancer” or “breast carcinoma” or “mammary cancer”, and “polymorphism” or “mutation” or “variant”. Additionally, other sources and reference lists of relevant reports were also checked to supplement the results of database searching.

### Selection criteria

The pre-designed criteria for each included study were as followed: with a case-control design; assessing the relationship between the *CD152* polymorphisms and BC susceptibility; stating sufficient data on genotype and/or allele frequencies of studied polymorphisms both in case and control groups for calculating odds ratios (ORs) with their corresponding 95% confidence intervals (95% CIs); and published in English or Chinese language. As for excluded publications, they met at least one of the following conditions: based on duplicated data; focusing on animals; and letters, commentaries, case report, review articles or conference abstracts. When the same group of study participants was incorporated into more than one report, the one with the largest sample size or most recently published was selected.

### Data extraction

Two independent reviewers were in charge of extracting primary information from all eligible articles using the same data table. Essential information abstracted contained first author's name, publication year, original country, ethnicity, genotyping method, number of cases and controls, genotype and/or allele frequencies in case and control groups, and *P* value for Hardy-Weinberg equilibrium (HWE) in controls. Any discrepancies over extracted data were settled through discussion between the two reviewers; if no consensus was reached through such approach, a third reviewer would be invited into the discussion.

### Statistical analysis

The intensity of the association between *CD152* polymorphisms and BC susceptibility was appraised through calculating pooled ORs and their 95% CIs. Heterogeneity between included studies was detected with *Q* test. *P* < 0.05 suggested significant heterogeneity, and the random-effects model was used; otherwise, the fixed-effects model was applied to assess the combined results. Sensitivity analysis was performed by omitting one study each time. Between-study publication bias was examined with both Begg's funnel plot and Egger's regression test. All these statistical analyses were completed with STATA 12.0 software (Stata Corporation, College Station, TX, USA).
